# Supplementary material for: Unraveling the mechanisms of deep-brain stimulation of the internal capsule in a mouse model
Source: Nat Commun. 2023 Sep 4;14:5385. doi: 10.1038/s41467-023-41026-x (PMC10477328; doi:10.1038/s41467-023-41026-x)
Supplement: Supplementary file 1 — Supplementary Information [file 41467_2023_41026_MOESM1_ESM.pdf]

Supplementary information supporting

van den Boom et al. "Unraveling the mechanisms of deep-brain stimulation of the internal capsule in a mouse model"

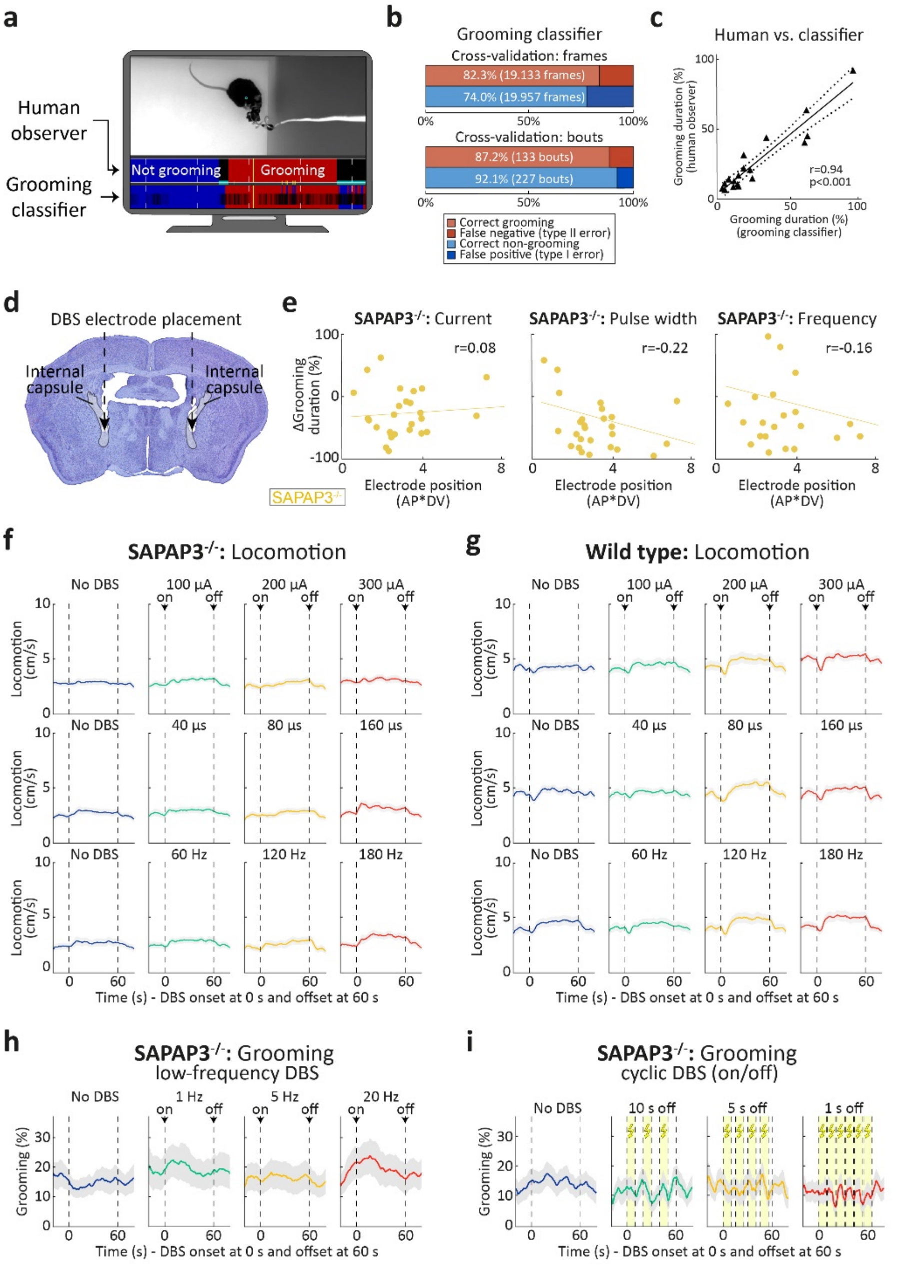

Supplementary Fig. 1 | IC-DBS electrode location did not correlate with grooming reduction and did not affect locomotion. a, Videos of behavior in the open field were used to train a grooming classifier using JAABA software[92]. Top row are behavioral assessments from a human observer, bottom row are predictions from the grooming classifier (red=grooming, blue=not grooming). **b**, Grooming classifier performance on single frames using k-fold cross-validation (true positive: 82.3%, true negative: 74.0%) (top). Grooming classifier performance on bouts (true positive: 87.2%, true negative: 92.1%) (bottom). **c**, Correlation of grooming scores from the human expert-observer versus the grooming classifier. **d**, Cresyl-violet staining with DBS electrodes in IC (gray) (representative example). **e**, IC-DBS electrode-position (two-dimensional anterior-posterior * dorsal-ventral) did not

correlate with grooming reduction during high-intensity DBS. Dots are individual animals. **f**, No DBS-induced changes in locomotion in SAPAP3$^{-/-}$. **g**, No DBS-induced changes in locomotion in WT. **h**, Low-frequency IC-DBS stimulation (1, 5, or 20 Hz) did not affect grooming in SAPAP3$^{-/-}$. **i**, Cyclic IC-DBS stimulation (DBS on for 10 s and off for 10, 5, or 1 s) did not affect grooming in SAPAP3$^{-/-}$. Data are mean±SEM. Source data are provided as a Source Data file. AP=anterior-posterior, DV=dorsal-ventral.

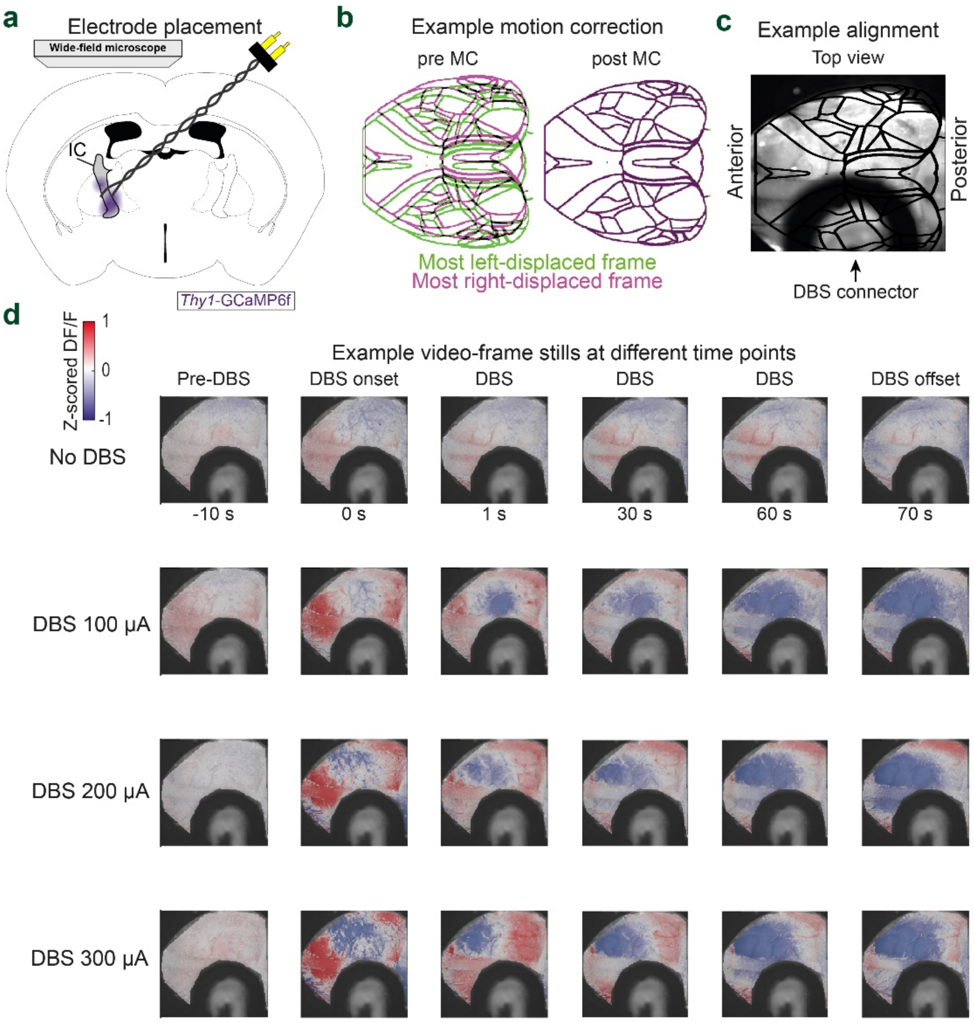

Supplementary Fig. 2 | Representative data for wide-field imaging experiments. a, Schematic of the wide-field setup with DBS-electrode placement in IC (gray) of *Thy1*-GCaMP6f mice (purple, *n*=5), projected on the Allen Reference Atlas - Mouse Brain[102]. Halo represents the modeled sphere of current spread around the DBS electrode tips. **b**, Motion correction (MC) aligns outline of individual wide-field video frames extracted from the Allen Mouse Brain Connectivity Atlas (right)[51], as depicted by overlap of maximum deviating frames (left: green; right: pink) after motion correction. **c**, Alignment of a wide-field video frame to the Allen mouse-brain atlas (example video frame). **d**, Video frames of calcium dynamics during different DBS parameters (rows) across different epochs (columns). MC=motion correction.

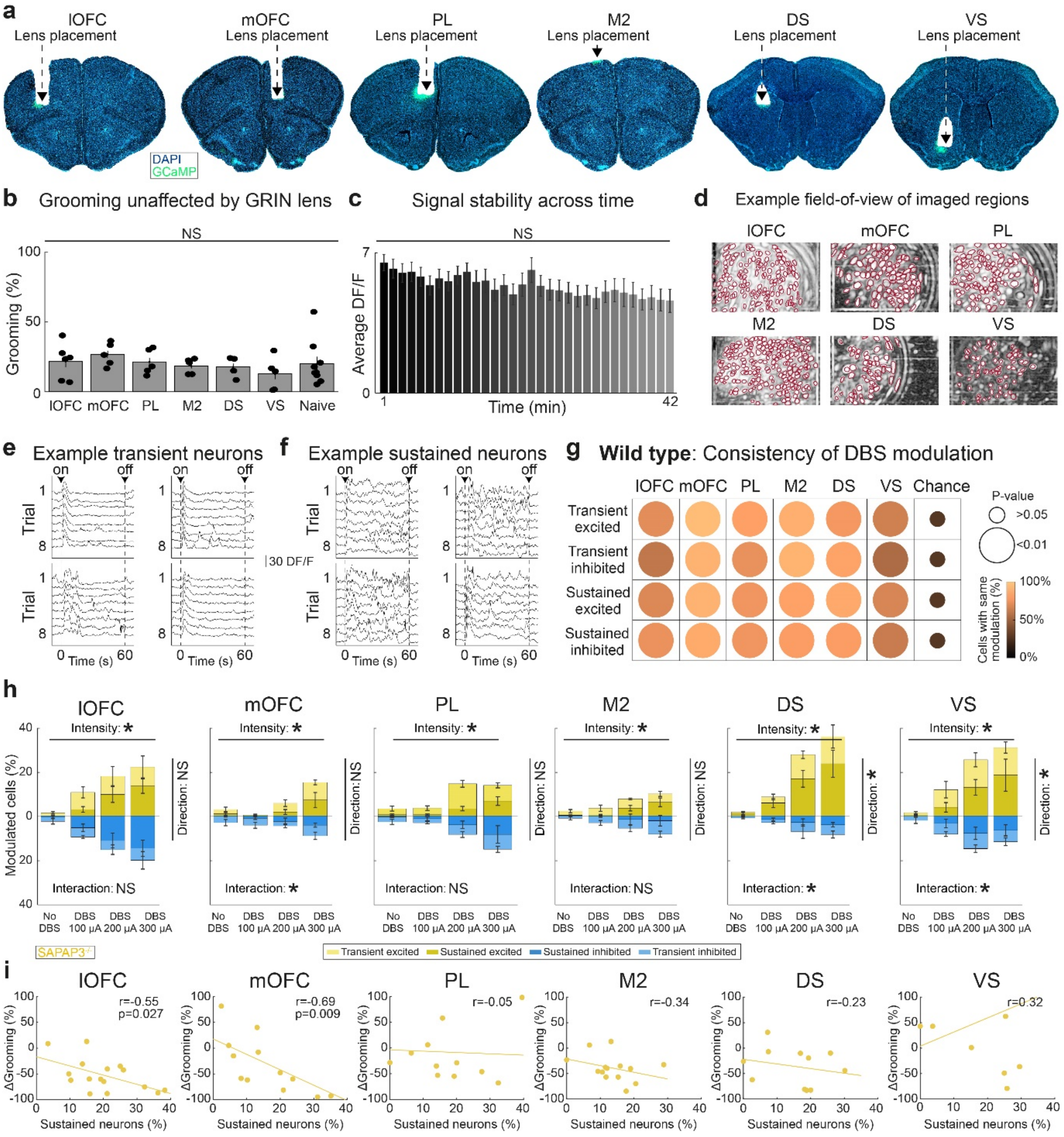

Supplementary Fig. 3 | Modulation of cortical and striatal regions in WT by IC-DBS. a, Histological verification of imaged regions (DAPI=blue, GCaMP6s=green). **b**, SAPAP3$^{-/-}$ grooming was unaffected by GRIN-lens implantation in all regions (SAPAP3$^{-/-}$: lOFC (*n*=6), mOFC (*n*=5), PL (*n*=5), M2 (*n*=5), DS (*n*=4), VS (*n*=5)). Naive animals were not implanted (*n*=9). **c**, Miniscope calcium-imaging signal was stable across the entire recording session and did not exhibit bleaching (*n*=21). **d**, Maximum-intensity projection per region with overlay neuron outlines (in red). **e**, Example of four transiently excited neurons. For each trial, the neurons showed exclusively increased fluorescence upon DBS onset. **f,** Example of four sustainedly excited neurons. During each trial, the neurons showed calcium dynamics throughout DBS. **g**, Similar to SAPAP3$^{-/-}$ (Fig. 3g), consistency of modulation of DBS was

significantly different from chance in all cortical and striatal regions recorded in WT. **h**, Similar to SAPAP3$^{-/-}$ (Fig 3f), we found dose-dependent recruitment of both excited and inhibited neurons by DBS (lOFC (*n*=5), PL (*n*=5), and M2 (*n*=4)). In other regions, we found differences in the number of neurons recruited by exciting or inhibiting their activity (mOFC (*n*=5), DS (*n*=5), and VS (*n*=4)). **i**, Grooming reduction correlated negatively with the number of sustainedly recruited neurons (expressed as percentage of all recorded neurons) in lOFC and mOFC of SAPAP3$^{-/-}$. Data are mean±SEM. Source data are provided as a Source Data file. *p<0.05, NS=not significant.

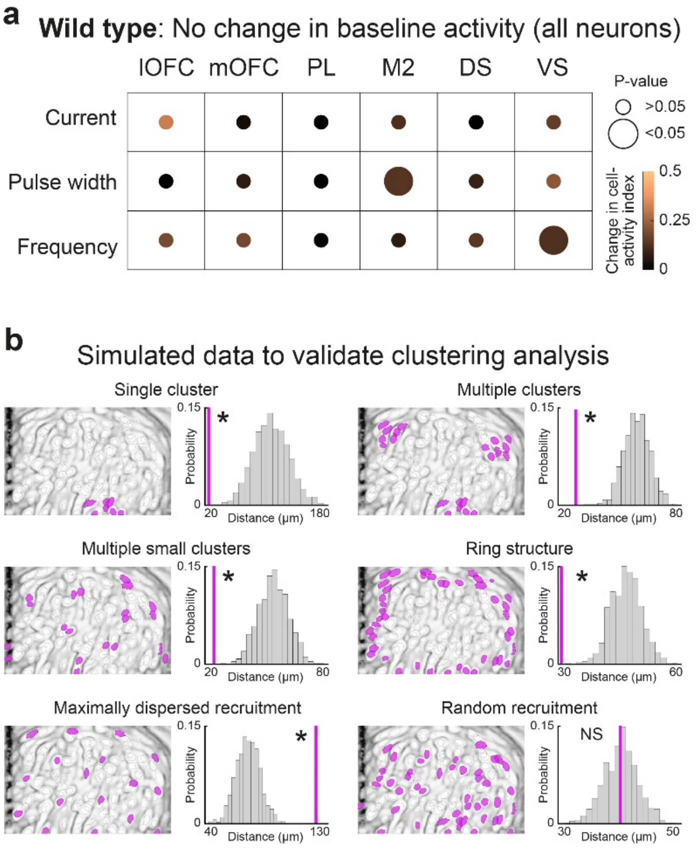

Supplementary Fig. 4 | Baseline activity in WT and validation of cell-clustering analysis. a, Similar to SAPAP3$^{-/-}$ (Fig. 4b), DBS did not alter cell-activity index in WT. **b**, Simulated data demonstrated detection of different forms of clustering and dispersiveness. By comparing the distance to the closest neighbor to chance (bootstrap), the analysis can detect clustering as a single cluster, multiple clusters, multiple small clusters, or ring structure. In addition, dispersed recruitment (neurons distributed at equal distance throughout the field-of-view) and truly random recruitment could be detected. Data are mean. Source data are provided as a Source Data file. **p*<0.05, NS=not significant.

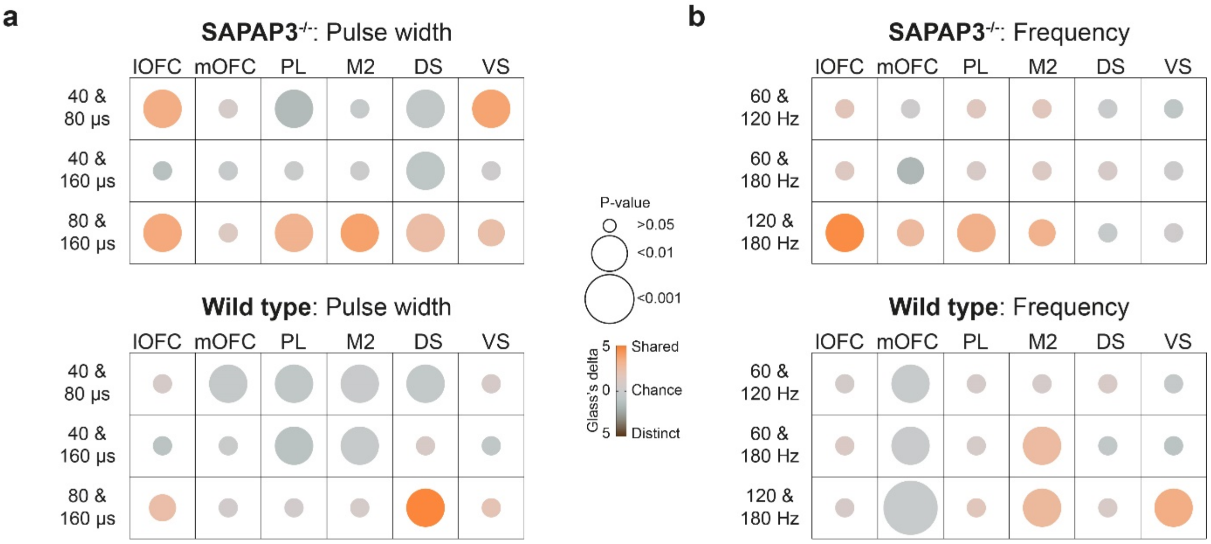

Supplementary Fig. 5 | DBS recruits similar neuron populations during medium- and high-intensity DBS. **a**, For the SAPAP3$^{-/-}$ pulse-width dose-response experiment, recruited neuron populations overlap significantly between 80 and 160 μs in almost all regions (top panel), but with little overlap in WT (bottom panel). **b**, Similar results as in panel b, but during the frequency dose-response experiment. Data are mean. Source data are provided as a Source Data file.

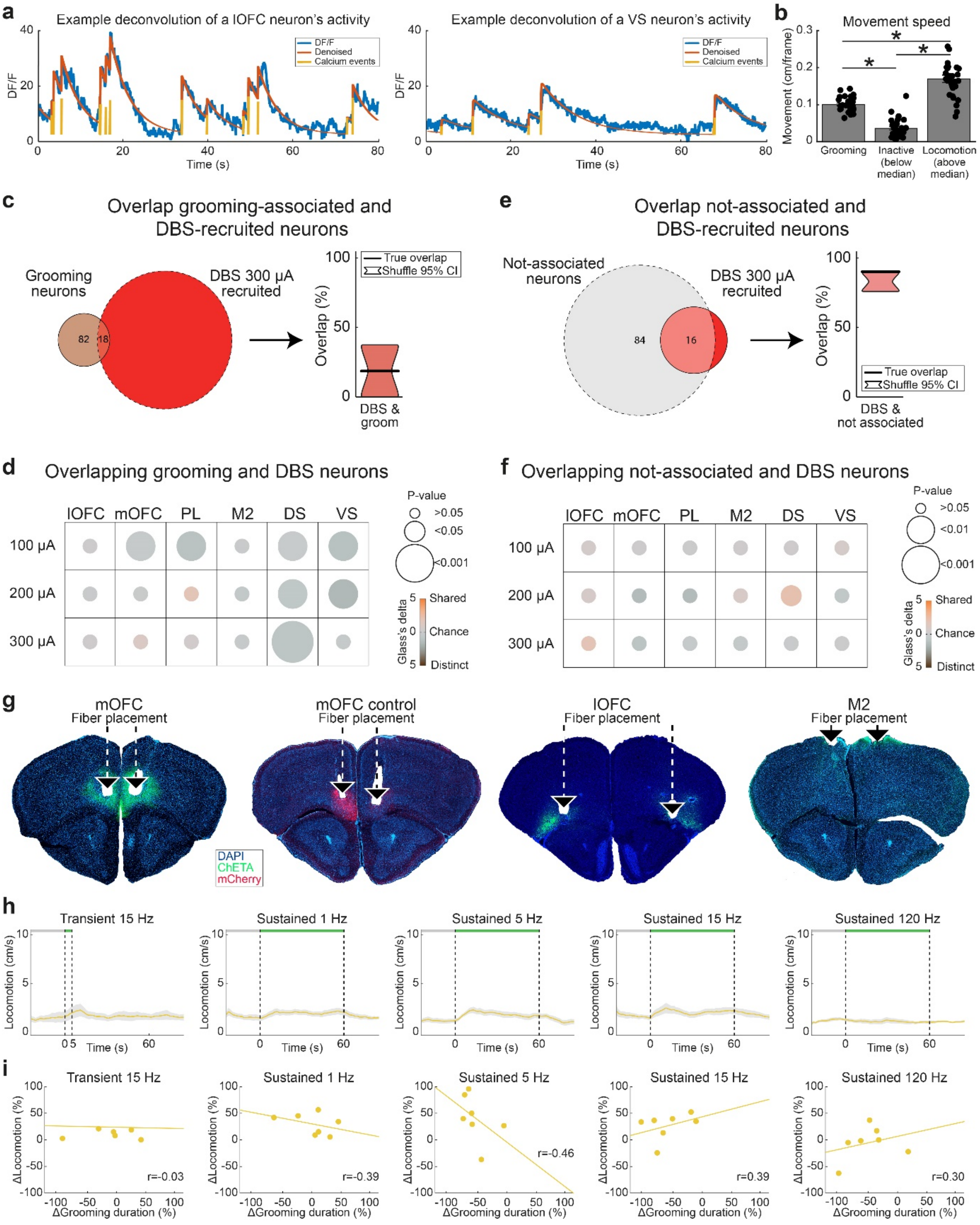

Supplementary Fig. 6 | Non-specific recruitment of behavior-associated neurons by DBS and lack of locomotion effects during photostimulation. a, Estimated calcium events (yellow) based on deconvolved trace (red) of fluorescent signal (blue) of a lOFC neuron (left) and a VS neuron (right). **b**, Average SAPAP3$^{-/-}$ movement during grooming, inactive epochs (below median), and locomotion (above median). Inactive and locomotion

epochs consist of remaining movement after grooming-movement subtraction. All groups differed significantly, indicating that below-median data predominantly tracks inactivity, whereas above-median data predominantly tracks locomotion (*n*=30). **c**, Overlap between grooming-associated neurons and DBS-recruited neurons (left) did not significantly differ from chance (bootstrap, right). **d**, DBS did not recruit grooming-associated neurons above chance (i.e., shared, overlapping neuron populations), or consistently below chance (i.e., distinct, unique neuron populations). **e**, Similar to panel c, but for not-associated neurons (i.e., not associated with grooming, inactivity, or locomotion). **f**, Similar to panel d, but for not-associated neurons. **g,** Histological verification of stimulated regions (DAPI=blue, ChETA=green, mCherry=red). **h**, Locomotion during optogenetic stimulation of mOFC did not change across different stimulation frequencies. **i**, No significant correlation between reduced grooming and locomotion was found for any mOFC-photostimulation frequency. Data are mean±SEM. Source data are provided as a Source Data file. CI=confidence interval, *p<0.05.
