# Supplementary material for: Unraveling the mechanisms of deep-brain stimulation of the internal capsule in a mouse model
Source: Nat Commun. 2023 Sep 4;14:5385. doi: 10.1038/s41467-023-41026-x (PMC10477328; doi:10.1038/s41467-023-41026-x)
Supplement: Supplementary file 3 — Reporting Summary [file 41467_2023_41026_MOESM3_ESM.pdf]

nature portfolio

Corresponding author(s): Bastijn van den Boom & Ingo Willuhn

Last updated by author(s): Jul 6, 2023

Reporting Summary

Nature Portfolio wishes to improve the reproducibility of the work that we publish. This form provides structure for consistency and transparency in reporting. For further information on Nature Portfolio policies, see our Editorial Policies and the Editorial Policy Checklist.

Statistics

For all statistical analyses, confirm that the following items are present in the figure legend, table legend, main text, or Methods section.

n/a	Confirmed	
☐	☒	The exact sample size (*n*) for each experimental group/condition, given as a discrete number and unit of measurement
☐	☒	A statement on whether measurements were taken from distinct samples or whether the same sample was measured repeatedly
☐	☒	The statistical test(s) used AND whether they are one- or two-sided<br>*Only common tests should be described solely by name; describe more complex techniques in the Methods section.*
☒	☐	A description of all covariates tested
☐	☒	A description of any assumptions or corrections, such as tests of normality and adjustment for multiple comparisons
☐	☒	A full description of the statistical parameters including central tendency (e.g. means) or other basic estimates (e.g. regression coefficient) AND variation (e.g. standard deviation) or associated estimates of uncertainty (e.g. confidence intervals)
☐	☒	For null hypothesis testing, the test statistic (e.g. *F*, *t*, *r*) with confidence intervals, effect sizes, degrees of freedom and *P* value noted<br>*Give P values as exact values whenever suitable.*
☒	☐	For Bayesian analysis, information on the choice of priors and Markov chain Monte Carlo settings
☒	☐	For hierarchical and complex designs, identification of the appropriate level for tests and full reporting of outcomes
☐	☒	Estimates of effect sizes (e.g. Cohen's *d*, Pearson's *r*), indicating how they were calculated

Our web collection on statistics for biologists contains articles on many of the points above.

Software and code

Policy information about availability of computer code

Data collection	Bonsai V2.5.3 (open-source): Behavioral video recording and controlling DBS, imaging, and opto<br>Encephalos software V1 (Caenotec): Wide-field imaging microscope control and imaging capture<br>miniscope DAQ V3.2 (open-source): Miniscope imaging microscope control and imaging capture
Data analysis	Bonsai V2.5.3 (open-source): Behavioral video tracking<br>FIJI 1.53 (open-source): Video reading software<br>Mouse Tracker 1.0 (open-source): Detailed frame-by-frame position information<br>JAABA 0.5.0 (open-source): Trained grooming classifier<br>NoRMCorre (open-source): Miniscope calcium imaging motion correction<br>CNMF-E (open-source): Miniscope calcium imaging spatial footprint and temporal activity extraction of neurons<br>Matlab 2016b & 2020b (Mathworks inc.): Preprocessing, plotting, and statistically testing of all data

For manuscripts utilizing custom algorithms or software that are central to the research but not yet described in published literature, software must be made available to editors and reviewers. We strongly encourage code deposition in a community repository (e.g. GitHub). See the Nature Portfolio guidelines for submitting code & software for further information.

Data

Policy information about availability of data

All manuscripts must include a data availability statement. This statement should provide the following information, where applicable:

- Accession codes, unique identifiers, or web links for publicly available datasets
- A description of any restrictions on data availability
- For clinical datasets or third party data, please ensure that the statement adheres to our policy

Source data are provided with this paper. The data that support the findings reported in this article are available on Open Science Framework https://osf.io/w7qte/. The statistical analyses generated from the data are available on Open Science Framework (https://osf.io/w7qte/). Raw data are available from the corresponding author upon reasonable request.

Human research participants

Policy information about studies involving human research participants and Sex and Gender in Research.

Reporting on sex and gender	N/A
Population characteristics	N/A
Recruitment	N/A
Ethics oversight	N/A

Note that full information on the approval of the study protocol must also be provided in the manuscript.

Field-specific reporting

Please select the one below that is the best fit for your research. If you are not sure, read the appropriate sections before making your selection.

☒ Life sciences ☐ Behavioural & social sciences ☐ Ecological, evolutionary & environmental sciences

For a reference copy of the document with all sections, see nature.com/documents/nr-reporting-summary-flat.pdf

Life sciences study design

All studies must disclose on these points even when the disclosure is negative.

Sample size	No sample size calculations were made. Sample sizes were based on previous literature (e.g., Shuman et al., 2020). We repeated the experiment three times (different deep-brain stimulation conditions with one stimulation always overlapping across experiments) to ensure our findings are reliable and not due to chance.
Data exclusions	No mice were excluded from the analyses.
Replication	Each animal was tested three times across three experimental days. We focused on findings that were replicated across experiments, which ensured a very high reliability.
Randomization	Mice were randomly assigned to a given imaging region. For each experiment, blocks of stimulation were randomly assigned to mice and varied across mice.
Blinding	We employed an automated grooming classifier that avoided manual interference with behavior annotations. Mice were grouped based on imaging region and analyzed using the same automated analyses scripts. Investigators were blinded to group allocation during data collection and analysis.

Reporting for specific materials, systems and methods

We require information from authors about some types of materials, experimental systems and methods used in many studies. Here, indicate whether each material, system or method listed is relevant to your study. If you are not sure if a list item applies to your research, read the appropriate section before selecting a response.

Materials & experimental systems

n/a	Involved in the study
☒	☐ Antibodies
☒	☐ Eukaryotic cell lines
☒	☐ Palaeontology and archaeology
☐	☒ Animals and other organisms
☒	☐ Clinical data
☒	☐ Dual use research of concern

Methods

n/a	Involved in the study
☒	☐ ChIP-seq
☒	☐ Flow cytometry
☒	☐ MRI-based neuroimaging

Animals and other research organisms

Policy information about studies involving animals; ARRIVE guidelines recommended for reporting animal research, and Sex and Gender in Research

Laboratory animals	SAPAP3 mutant (C57Bl/6J backbone), wild-type littermates, and Thy1-GCaMP6f mice. Both sexes were used. Age range 2-8 months.
Wild animals	No wild animals were used.
Reporting on sex	Both sexes were used. Similar to our previous work (Pinhal et al 2018), we found not indication of sex differences on grooming.
Field-collected samples	No field-collected samples were used.
Ethics oversight	Animal Ethical Committee of the Royal Netherlands Academy of Arts and Sciences<br>Centrale Commissie Dierproven

Note that full information on the approval of the study protocol must also be provided in the manuscript.
