# Supplementary material for: Unraveling the mechanisms of deep-brain stimulation of the internal capsule in a mouse model
Source: Nat Commun. 2023 Sep 4;14:5385. doi: 10.1038/s41467-023-41026-x (PMC10477328; doi:10.1038/s41467-023-41026-x)
Supplement: Supplementary file 4 — Source Data [file 41467_2023_41026_MOESM4_ESM.zip › figure1_info.docx]

Figure1.mat contains data including grooming behavior during DBS (grooming) and movement behavior during DBS (movement).

Data are split per genotype (SAPAP3 KO and wild-type littermates) and DBS condition (current, pulse width, frequency, cyclic, low frequency).

Grooming: animal names (animal), mean grooming during no DBS (columns: pre DBS, during DBS) (groom0), mean grooming during low DBS (columns: pre DBS, during DBS) (groom1), mean grooming during medium DBS (columns: pre DBS, during DBS) (groom2), mean grooming during high DBS (columns: pre DBS, during DBS) (groom3), percentage difference in grooming reduction during no DBS (dif_groom0), percentage difference in grooming reduction during low DBS (dif_groom1), percentage difference in grooming reduction during medium DBS (dif_groom2), percentage difference in grooming reduction during high DBS (dif_groom3), mean dorsal-ventral coordinates of both bilateral electrodes according to the Paxinos brian atlas (paxi_mean_DV), mean anterior-posterior coordinates of both bilateral electrodes according to the Paxinos brian atlas (paxi_mean_AP), combined measure of both mean DV and AP coordinates (paxi_APxDV)

Movement: animal names (animal), mean movement during no DBS (columns: pre DBS, during DBS) (move0), mean movement during low DBS (columns: pre DBS, during DBS) (move1), mean movement during medium DBS (columns: pre DBS, during DBS) (move2), mean movement during high DBS (columns: pre DBS, during DBS) (move3)
