# Supplementary material for: Unraveling the mechanisms of deep-brain stimulation of the internal capsule in a mouse model
Source: Nat Commun. 2023 Sep 4;14:5385. doi: 10.1038/s41467-023-41026-x (PMC10477328; doi:10.1038/s41467-023-41026-x)
Supplement: Supplementary file 4 — Source Data [file 41467_2023_41026_MOESM4_ESM.zip › figure2_info.docx]

Figure2.mat contains data including widefield calcium-imaging signal across the entire dorsal cortex (dorsal_cortex), per cortical region (per_region), and comparing cortical regions (compare_regions).

Data are split per DBS condition (current, pulse width, frequency).

Dorsal_cortex: mean activity before DBS (rows: animals; columns: no DBS, low, medium, high) (mean_pre), mean activity during DBS (rows: animals; columns: no DBS, low, medium, high) (mean_DBS)

Per_region: [data are split per region (PFC, SS, RSP, VIS)] mean activity before DBS (rows: animals; columns: no DBS, low, medium, high) (mean_pre), mean activity during DBS (rows: animals; columns: no DBS, low, medium, high) (mean_DBS)

Compare_regions: mean activity before DBS (rows: animals; columns: PFC, SS, RSP, VIS) (mean_pre), mean activity during DBS (rows: animals; columns: PFC, SS, RSP, VIS) (mean_DBS)
