# Supplementary material for: Unraveling the mechanisms of deep-brain stimulation of the internal capsule in a mouse model
Source: Nat Commun. 2023 Sep 4;14:5385. doi: 10.1038/s41467-023-41026-x (PMC10477328; doi:10.1038/s41467-023-41026-x)
Supplement: Supplementary file 4 — Source Data [file 41467_2023_41026_MOESM4_ESM.zip › figure3_info.docx]

Figure3.mat contains data including baseline grooming behavior after GRIN lens implantation (grooming_GRIN_lens), stability of calcium imaging signal (signal_stability), correlation between grooming reduction and sustained neurons (groom_sust_correl), and modulation of neurons by DBS (DBS_mod)

Grooming_GRIN_Lens: animal names (animals), region of imaging (region), calculated lesion size induced by the GRIN lens (volume_lesion_by_GRIN), mean grooming behavior during no DBS (groom0_mean)

Signal_stability: animal names (animals), imaging region (region), mean calcium signal in 1-minute bins (signal)

Groom_sust_correl: [data are split per region (DS, lOFC, M2, mOFC, PL, VS)] animal names (animals), DBS condition (condition), percentage of sustained neurons recruited by high DBS (sust3_perc), grooming reduction during high DBS (groom_red)

DBS_mod: [data are split per genotype (SAPAP3 KO and wild-type littermates), per region (DS, lOFC, M2, mOFC, PL, VS), and DBS condition (current, pulse width, frequency)] animal names (animals), number of neurons recorded (n_cells), percentage of recruited neurons by DBS (rows: no DBS, low, medium, high; columns: transient increase, sustained increase, transient decrease, sustained decrease, not modulated) (responsive_perc), percentage of consistent recruitment by DBS (rows: transient increase, sustained increase, transient decrease, sustained decrease; columns: consistent, not consistent)
