# Supplementary material for: Unraveling the mechanisms of deep-brain stimulation of the internal capsule in a mouse model
Source: Nat Commun. 2023 Sep 4;14:5385. doi: 10.1038/s41467-023-41026-x (PMC10477328; doi:10.1038/s41467-023-41026-x)
Supplement: Supplementary file 4 — Source Data [file 41467_2023_41026_MOESM4_ESM.zip › figure4_info.docx]

Figure4.mat contains data including baseline activity measure (baseline_activity), spatiotemporal correlations between neurons (spatiotemporal_correl), and closest distance to neighboring neurons (distance).

Data are split per genotype (SAPAP3 KO and wild-type littermates), DBS condition (current, pulse width, frequency), and region (DS, lOFC, M2, mOFC, PL, VS).

Baseline_activity: animal names (animals), mean baseline activity during no DBS (mean_0), mean baseline activity during high DBS (mean_3)

Spatiotemporal_correl: animal names (animals), mean distance to neighbor neurons in nine spatial bins (mean_dist), mean correlation to neighbor neurons in same nine spatial bins (mean_corr). Nine bins: 0-20 20-30 30-40 40-50 50-60 60-70 70-80 80-90 90-100 µm

Distance: animal names (animals), distance to closest neighbor neuron during low DBS (closest_1) , distance to closest neighbor neuron during medium DBS (closest_2) , distance to closest neighbor neuron during high DBS (closest_3)
