# Supplementary material for: Unraveling the mechanisms of deep-brain stimulation of the internal capsule in a mouse model
Source: Nat Commun. 2023 Sep 4;14:5385. doi: 10.1038/s41467-023-41026-x (PMC10477328; doi:10.1038/s41467-023-41026-x)
Supplement: Supplementary file 4 — Source Data [file 41467_2023_41026_MOESM4_ESM.zip › figure5_info.docx]

Figure5.mat contains data including dose-dependent recruitment of neurons by DBS (dose_response), overlap between DBS recruited neurons across DBS conditions (venn_overlap), and excitation/inhibition balance (E_I_balance).

Data are split per genotype (SAPAP3 KO or wild-type littermates), DBS condition (current, pulse width, frequency), and region (DS, lOFC, M2, mOFC, PL, VS).

dose_response: animal names (animals), percentage of DBS recruited neurons (rows: no DBS, low, medium, high)

venn_overlap: animal names (animals), percentage of neurons overlapping (columns: only low DBS, low and medium, only medium, medium and high, only high, high and low, low and medium and high, no overlap) (venn_overlap_perc)

E_I_balance: animal names (animals), percentage of excitation inhibition (columns: no DBS, low, medium, high) (E_I_perc)
