# Supplementary material for: Unraveling the mechanisms of deep-brain stimulation of the internal capsule in a mouse model
Source: Nat Commun. 2023 Sep 4;14:5385. doi: 10.1038/s41467-023-41026-x (PMC10477328; doi:10.1038/s41467-023-41026-x)
Supplement: Supplementary file 4 — Source Data [file 41467_2023_41026_MOESM4_ESM.zip › figure6_info.docx]

Figure6.mat contains data including percentage of behavioral neurons (beh_neurons: grooming, movement, both, not-modulated), behavioral neurons modulated by DBS (DBS_beh_neurons: low, medium, high), percentage DBS reduced grooming neurons (DBS_red_groom_perc), overlap between identity of DBS (low, medium, high) and behavioral neurons (grooming, not-modulated) (venn_overlap), grooming behavior during optogenetic stimulation (opto_grooming: before opto, during opto), movement during optogenetic stimulation (opto_movement: before opto, during opto), and movements binned into different speeds (movement_speed).

Data are split per genotype (only SAPAP3 KO), DBS condition (current, pulse width, frequency, optogenetic stimulation), and region (DBS: DS, lOFC, M2, mOFC, PL, VS; opto: lOFC, M2, mOFC, mOFC_blocked, mOFC_ctrl).

beh_neurons: animal names (animals), percentage grooming neurons (groom_perc), percentage movement neurons (move_perc), percentage both neurons (both_perc), percentage not-modulated neurons (none_perc)

DBS_beh_neurons: animal names (animals), percentage of grooming neuron modulated by DBS (columns: low, medium, high) (DBS_groom_perc), percentage of not-modulated neurons modulated by DBS low, medium, high (columns) (DBS_none_perc)

DBS_red_groom_perc: animal names (animals), percentage reduction in grooming neurons during DBS (groom_red_perc)

venn_overlap: animal names (animals), percentage overlap between identity of DBS (columns: low, medium, high) and grooming neuron (overlap_groom), percentage overlap between identify of DBS (columns: low, medium, high) and not-modulated neurons (overlap_none)

opto_grooming: animal names (animals), percentage grooming behavior before optogenetic stimulation (columns: transient 15 Hz, sustained 1, 5, 15, 120 Hz) (grooming_pre), percentage grooming behavior during optogenetic stimulation (columns: transient 15 Hz, sustained 1, 5, 15, 120 Hz) (grooming_opto)

opto_movement: animal names (animals), percentage movement before optogenetic stimulation (columns: transient 15 Hz, sustained 1, 5, 15, 120 Hz) (movement_pre), percentage movement during optogenetic stimulation (columns: transient 15 Hz, sustained 1, 5, 15, 120 Hz) (movement_opto)

movement_speed: animal names (animals), mean speed during grooming (mean_moveGroom), mean speed during inactivity (mean_moveLow), mean speed during locomotion (mean_moveHigh)
